# A comprehensive analysis of CD47 expression in various histological subtypes of soft tissue sarcoma: exploring novel opportunities for macrophage-directed treatments

**DOI:** 10.1007/s00432-024-05661-1

**Published:** 2024-03-17

**Authors:** Iva Benesova, Linda Capkova, Andrej Ozaniak, Pavel Pacas, Katerina Kopeckova, Dominika Galova, Robert Lischke, Tomas Buchler, Zuzana Ozaniak Strizova

**Affiliations:** 1 Department of Immunology, Second Faculty of Medicine, Charles University and University Hospital Motol, V Uvalu 84, 150 06, Prague 5, Czech Republic; 2 Department of Pathology and Molecular Medicine, Second Faculty of Medicine, Charles University and University Hospital Motol, V Uvalu 84, 150 06, Prague, Czech Republic; 3 Third Department of Surgery, 1st Faculty of Medicine, Charles University and University Hospital Motol, V Uvalu 84, 150 06, Prague, Czech Republic; 4 Department of Oncology, Second Faculty of Medicine, Charles University and University Hospital Motol, V Uvalu 84, 150 06, Prague, Czech Republic

**Keywords:** Soft tissue sarcoma, CD47, Immunotherapy, Immune checkpoint inhibitors, Macrophages

## Abstract

**Purpose:**

The CD47 molecule, often referred to as the “do not eat me” signal, is frequently overexpressed in tumor cells. This signaling pathway limits phagocytosis by macrophages. Our objective was to determine CD47 abundance in various soft tissue sarcomas (STS) to investigate whether it could serve as a potential evasion mechanism for tumor cells. Additionally, we aimed to assess the prognostic value of CD47 expression by examining its association with different clinicopathological factors. This study aimed to elucidate the significance of CD47 in the context of emerging anti-tumor targeting approaches.

**Methods:**

In this retrospective study, formalin-fixed paraffine-embedded (FFPE) tumor tissues of 55 treatment-naïve patients were evaluated by immunohistochemistry for the abundance of CD47 molecule on tumor cells. The categorization of CD47 positivity was as follows: 0 (no staining of tumor cells), 1 + (less than 1/3 of tumor area positive), 2 + (between 1/3 and 2/3 of tumor area positive), and 3 + (more than 2/3 of tumor area positive for CD47). Next, we compared CD47 abundance between different tumor grades (G1–3). We used Kaplan–Meier survival curves with log-rank test to analyze the differences in survival between patients with different CD47 expression. Moreover, we performed Cox proportional hazards regression model to evaluate the clinical significance of CD47.

**Results:**

CD47 is widely prevalent across distinct STS subtypes. More than 80% of high grade undifferentiated pleiomorphic sarcoma (UPS), 70% of myxofibrosarcoma (MFS) and more than 60% of liposarcoma (LPS) samples displayed a pattern of moderate-to-diffuse positivity. This phenomenon remains consistent regardless of the tumor grade. However, there was a tendency for higher CD47 expression levels in the G3 group compared to the combined G1 + G2 groups when all LPS, MFS, and UPS were analyzed together. No significant associations were observed between CD47 abundance, death, and metastatic status. Additionally, high CD47 expression was associated with a statistically significant increase in progression-free survival in the studied cohort of patients.

**Conclusion:**

This study highlights the potential of the CD47 molecule as a promising immunotherapeutic target in STS, particularly given its elevated expression levels in diverse sarcoma types. Our data showed a notable trend linking CD47 expression to tumor grade, while also suggesting an interesting correlation between enhanced abundance of CD47 expression and a reduced hazard risk of disease progression. Although these findings shed light on different roles of CD47 in STS, further research is crucial to assess its potential in clinical settings.

## Introduction

Soft tissue sarcomas (STS) are a heterogeneous group of rare tumors consisting of more than 100 histological subtypes (Sbaraglia et al. 2021). For the management of localized tumors, the cornerstone treatment modality is microscopic radical (R0) surgical resection, with the possibility of integrating neoadjuvant and adjuvant radiotherapy and chemotherapy to optimize clinical outcomes. Regardless of multimodal therapy, 40–50% of patients develop metastases (Li et al. 2020). Anthracycline-based chemotherapy is the mainstay of treatment for patients with advanced or metastatic STS, but the response rate remains notably low, at roughly 20%. Furthermore, patient survival with generalized disease is poor, ranging approximately from 12 to 15 months (Banks and D’Angelo 2022; Meyer and Seetharam 2019). Immunotherapy, particularly the advent of immune checkpoint inhibitors (ICI), has revolutionized the treatment paradigm for multiple cancer types, serving as a transformative milestone in the field (Lee et al. 2022). ICI represent therapeutic agents that selectively target immune checkpoint molecules, such as PD-1, LAG-3, and CTLA-4, and thus, modulate immune response and restore anti-tumor activity.

While it is generally believed that STS have low immune cell infiltration, this is not entirely accurate for all STS subtypes and patients (Petitprez et al. 2020; Toulmonde et al. 2020). Although immune checkpoint molecules have been detected in various STS (Kelany et al. 2021; Klaver et al. 2020; Orth et al. 2020), clinical trials of these targets have not shown promising results (Banks & D’Angelo 2022). The objective response rate in patients with STS following treatment with the combination of nivolumab (anti-PD-1 antibody) and ipilimumab (anti-CTLA-4 antibody) was found to be 16%, which was similar to the response rate obtained with the conventional chemotherapy drug doxorubicin (overall response rate of 14%) (D’Angelo et al. 2018; Judson et al. 2014). Some histological subtypes are partially sensitive to ICI. For instance, promising outcomes have been reported in undifferentiated pleomorphic sarcoma (UPS), dedifferentiated liposarcoma (DDLPS), and synovial sarcoma, suggesting their potential as favorable candidates for ICI therapy. This was documented in a phase 2 study, where a remarkable response rate of 40% was observed in UPS following pembrolizumab treatment. Liposarcomas (LPS) demonstrated a comparatively lower yet still noteworthy response rate of 20% (Tawbi et al. 2017).

Further analysis revealed that the responders had higher baseline levels of PD-L1^+^ macrophages, tumor cells, regulatory T cells, and effector memory cytotoxic T cells (Keung et al. 2020). Additionally, a positive correlation has been observed between the intratumoral presence of tertiary lymphoid structures (TLS) and treatment outcomes, and thus, TLS may serve as a novel predictive biomarker (Italiano et al. 2022; Petitprez et al. 2020). These observations highlight the need for histological subtype-driven and patient-specific STS treatments. Moreover, current clinical trials combine immunotherapeutic drugs with chemotherapy, radiotherapy, and targeted therapy or test the effects of multiple ICI to increase the treatment effectiveness (Banks and D’Angelo 2022).

While the majority of current immunotherapies primarily concentrate on targeting and enhancing the effector functions of T cells, it is crucial to acknowledge that within the tumor microenvironment (TME), other immune cells, such as macrophages or NK cells, possess significant potential in the treatment of metastatic diseases (Ben-Shmuel et al. 2020; Mantovani et al. 2022). Therefore, there is an urgent need for the exploration of novel immunotherapeutic targets, suitable for both monotherapy and combination therapy to advance the development of more effective treatment approaches in STS.

Macrophages appear to be a promising immune cell subset for therapeutic interventions because they outnumber lymphocytes in nearly all histological subtypes of STS (Dancsok et al. 2020; Tsagozis et al. 2019). They act as double-edged swords in cancer because of their varied roles, which include phagocytosis, activation of other immune cells, and destruction of cancer cells, but also creation of immunosuppressive microenvironment, release of growth factors, induction of angiogenesis, and promotion of metastatic spread of tumors. (Mantovani et al. 2022; Strizova et al. 2023). Macrophages express a variety of immune checkpoint molecules such as PD-L1, PD-L2, TIM-3, and VISTA, which further fuel the immunosuppressive milieu within the TME (Brom et al. 2022). Similarly, macrophage functions are modulated by myeloid checkpoint regulators that upon binding their respective partners inhibit phagocytosis and induce immunosuppressive functions (Mantovani et al. 2022). Among the most studied myeloid checkpoint molecules are signal regulatory protein alpha (SIRP-α) and its ligand CD47. SIRP-α is widely expressed in myeloid cells, these include macrophages, which play a pivotal role in the anti-tumor immune response. Signaling through this receptor leads to the inhibition of cytoskeletal rearrangement via deactivation of myosin II at the phagocytic synapsis, and therefore restricts phagocytosis (Z. Li et al. 2021). CD47, also known as integrin-associated protein (IAP), is a “do not eat me” molecule that prevents phagocytosis and, thus, maintains homeostasis and minimizes the risk of autoimmune reactions. Apart from SIRP-α, CD47 interacts with thrombospondin 1 (TSP1) and various integrins (such as α2β1and αvβ3). Its binding to TSP1 regulates migration, angiogenesis, adhesion, apoptosis, and cell proliferation, while interaction with integrins maintains the migration of smooth cells and platelet activation (Hai et al. 2022; Hayat et al. 2020).

The indirect effects of CD47 signaling on other immune cells includes decreased tumor antigen presentation due to inhibition of phagocytosis, thus limiting T-cell activation. However, accumulating evidence emphasizes the direct immunoregulatory effects of CD47. Particularly, CD47 signaling modulates T-cell differentiation and survival, and restricts NK cell activation (Nath et al. 2022). Therefore, the anti-CD47 blockade might have a much broader effect than initially anticipated. Besides that, binding of anti-CD47 antibodies on tumor cells provides a strong “eat me” signal, which results in more efficient phagocytosis, and thereby it allows better presentation of tumor antigens with subsequent T-cell activation. Moreover, antibody-dependent cell-mediated cytotoxicity performed by macrophages (ROS, RNS), NK cells (perforin and granzymes), and neutrophils (trogoptosis) further enhances the immunotherapeutic potential (Duijn et al. 2022; Hai et al. 2022). Altogether, anti-CD47 therapy appears to be a worthy candidate for the treatment of solid cancers.

The objective of the present study was to investigate the abundance of CD47 molecule in multiple histological subtypes of STS to deeper understand the pathogenesis of these rare diseases and to guide the future therapeutic modalities for the treatment of patients with STS. Additionally, we explored the presence of CD47 at low and high tumor grades and assessed the clinical relevance of this molecule in our retrospective cohort.

## Methods

### Patient cohort

This retrospective study consisted of 55 treatment-naïve patients with primary STS that underwent surgical resection at the Third Department of Surgery, First Faculty of Medicine, Charles University and University Hospital Motol between February 2016 and March 2021. This study was approved by the Ethics Committee for Multi-Centric Clinical Trials of the University Hospital Motol (Ref. EK-189/20) and was conducted following the World Medical Association Declaration of Helsinki. Table 1 summarizes the clinicopathological characteristics of the patients with diverse STS subtypes included in this study. The tumor grade (G) was determined by an experienced pathologist using formalin-fixed paraffin-embedded (FFPE) tumor tissue.

**Table 1:** Clinicopathological characteristics of patients with soft tissue sarcoma (STS)

	*n* = 55 (%)
Age	
Interquartile range	62–78
Sex	
Female	26 (47)
Male	29 (53)
Grade	
G1	10 (18)
G2	5 (9)
G3	40 (73)
Metastatic disease	
No	36 (65)
Yes	19 (35)
Histology	
Undifferentiated pleiomorphic sarcoma (UPS)	15 (27)
Myxofibrosarcoma (MFS)	10 (18)
Liposarcoma (LPS)	22 (40)
Dedifferentiated liposarcoma (DDLPS)	10 (45)
Pleiomorphic liposarcoma (PLPS)	5 (23)
Myxoid liposarcoma (MLPS)	4 (18)
Well-differentiated liposarcoma (WDLPS)	3 (14)
Pleiomorphic rhabdomyosarcoma	3 (5)
Angiosarcoma	1 (2)
Leiomyosarcoma	1 (2)
Sarcoma not otherwise specified	1 (2)
Spindle cell sarcoma	1 (2)
Synovial sarcoma	1 (2)

### Immunohistochemistry

**Fig. 1:**
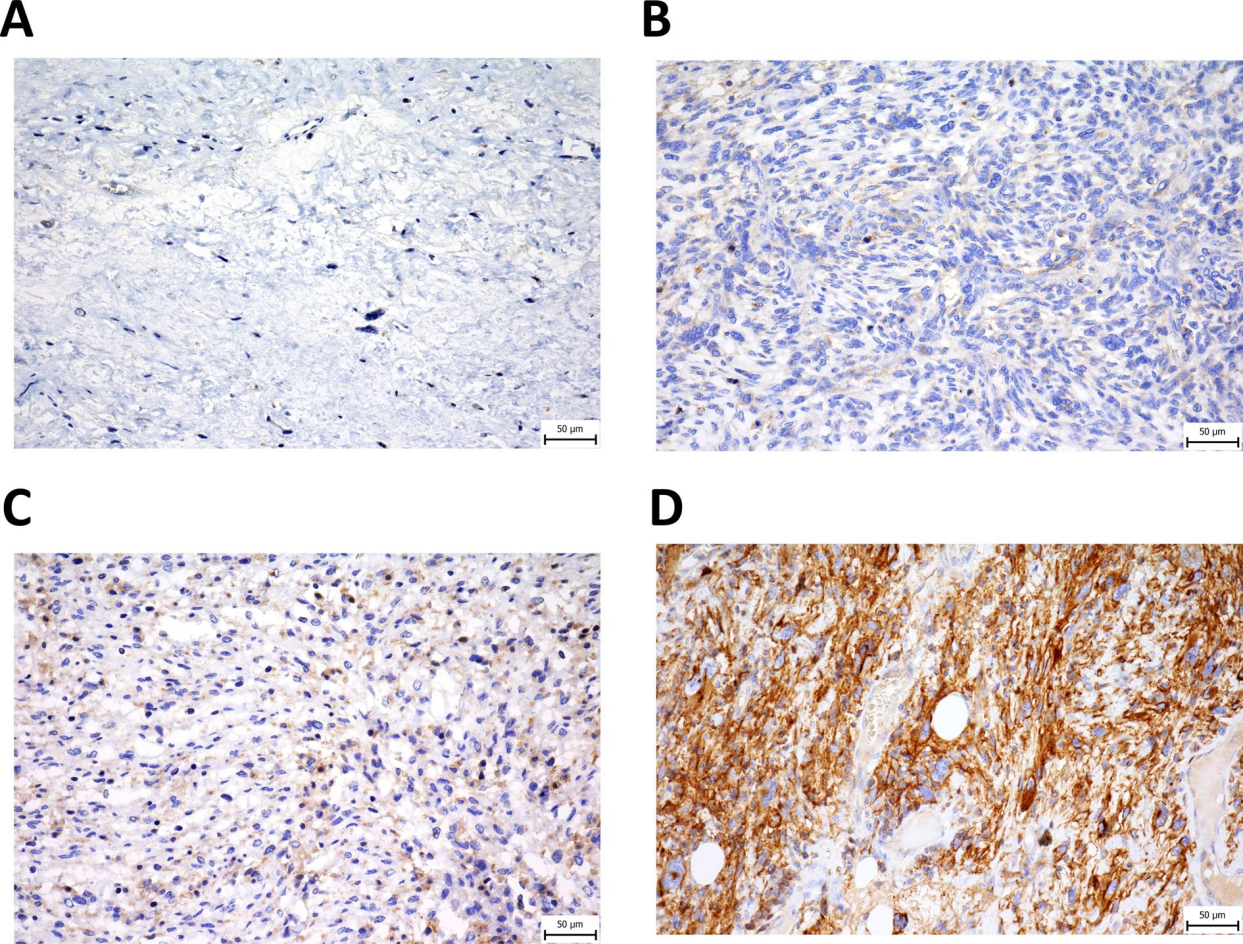
Representative example of CD47 expression in soft tissue sarcomas. **A** leiomyosarcoma 0; no staining of tumor cells, **B** undifferentiated pleiomorphic sarcoma 1 + ; less than 1/3 of the total tumor area positive (focal positivity), (**C**) dedifferentiated liposarcoma 2 + ; 1/3 – 2/3 of the total tumor area positive (moderate positivity) and **D** myxofibrosarcoma 3 + ; more than 2/3 of the total tumor area positive (diffuse positivity). Image magnification was 40 × and scale bars indicate 50 μm

Immunohistochemical analysis was performed as described previously (Ozaniak et al. 2022). Briefly, the FFPE tissue blocks were sectioned at 3 µm thickness. Next, the tissue slides were stained with a polyclonal anti-CD47 antibody (PA5-80435, Thermo Fisher Scientific, Massachusetts, USA). The diagnostic accuracy and validity of the slides were meticulously evaluated by a sarcoma specialized pathologist and each tissue sample was assessed and scored manually. To quantify the outcomes of the staining and mitigate potential subjective biases, an intra-rater reliability test was conducted. Cytoplasmic and membranous staining of the tumor cells was considered positive. The expression level of CD47 was classified on the following scale: 0, no staining of tumor cells (negative); 1 + , less than 1/3 of the total tumor area positive (focal positivity); 2 + , 1/3 – 2/3 of the total tumor area positive (moderate positivity); and 3 + , more than 2/3 of the total tumor area positive (diffuse positivity) (Fig. 1).

### Statistical analysis

The Shapiro–Wilk test was performed to test the data distribution for normality. Thereafter, the non-parametric Mann–Whitney U test was used to compare CD47 expression between the G1 + G2 and G3 groups. To analyze the association between clinical variables and CD47 expression, a chi-square test for independence was performed. We distinguished between patients who had metastases before the sampling and patients who developed metastases after the sampling. The Kaplan–Meier survival curve depicted differences in overall survival (OS), metastasis-free survival, and progression-free survival (PFS). The OS was defined as the interval between surgery and death. Metastasis-free survival was time period between surgery and the development of metastases or death. PFS was defined as the period between surgery and disease recurrence, metastasis development or death. The log-rank test and subsequent multiple comparisons tests with Bonferroni-corrected α-value were performed to analyze the significance. Survival analysis was performed on the entire cohort, as comparisons according to histological subtypes resulted in a low number of individuals in each group. Additionally, Cox proportional hazards regression analysis was performed to assess the prognostic relevance of CD47 expression for OS and PFS. The statistical analyses and data visualization were performed in GraphPad Prism 10.0.3 or R studio (package ggplot2) and *p* ≤ 0.05 was considered significant.

## Results

### CD47 exhibits moderate to high expression among STS regardless of the tumor grade

**Fig. 2:**
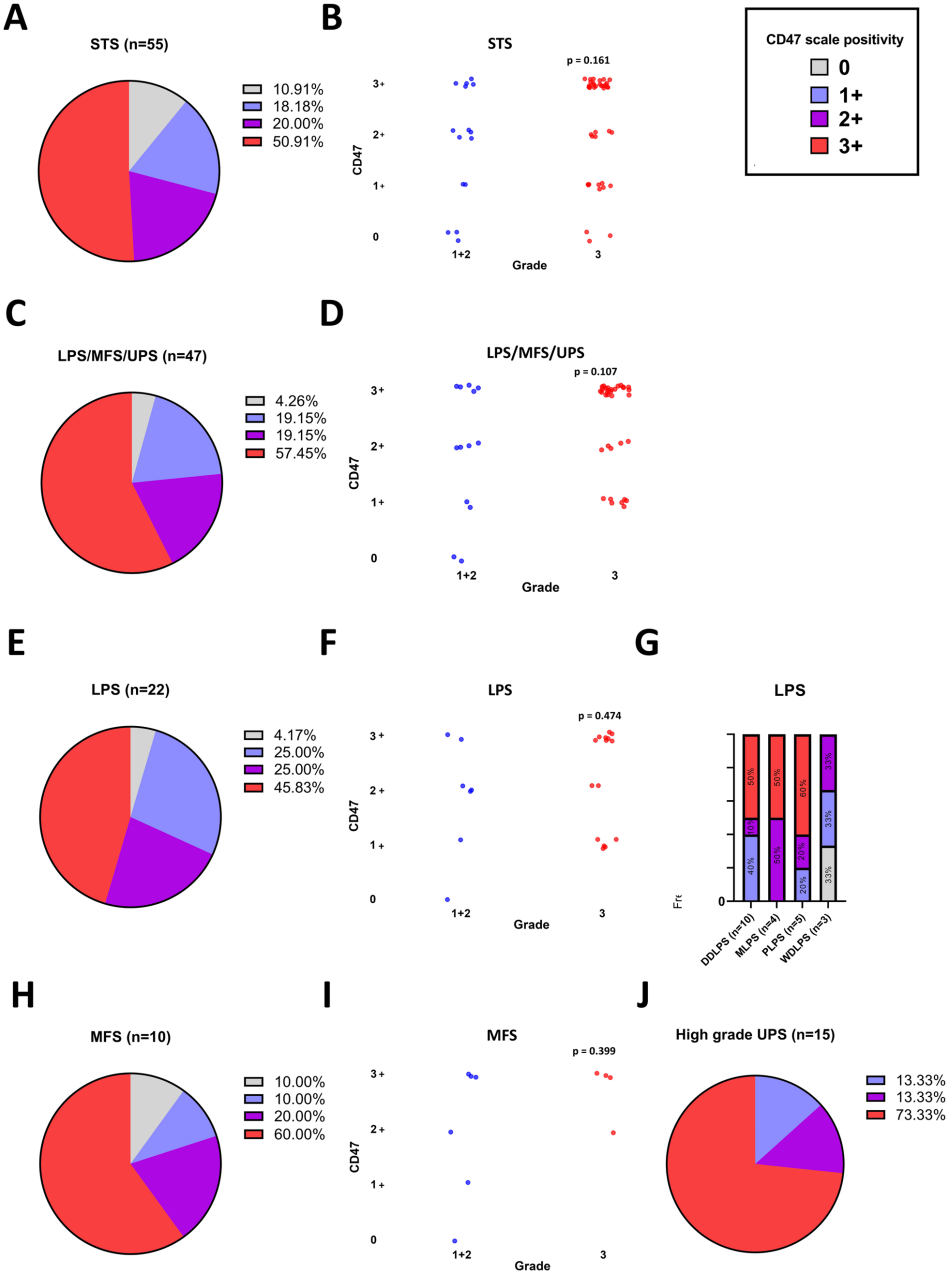
Expression of CD47 molecule among various histological subtypes of soft tissue sarcoma (STS). **A** CD47 scale positivity in (*n* = 55) and **B** frequency of CD47 scale positivity among grade (G)1 + G2 and G3. **C** CD47 scale positivity and **D** frequency in liposarcoma (LPS), myxofibrosarcoma (MFS) and undifferentiated pleiomorphic sarcoma (UPS) (*n* = 47). **E** CD47 scale positivity (*n* = 22) and **F** its frequency among G1 + G2 and G3 in LPS. **G** CD47 scale positivity in LPS divided according to histological subtypes. **H** frequency of CD47 scale positivity in myxofibrosarcoma (MFS) (*n* = 11) and **I** its frequency between G1 + G2 and G3. **J** CD47 scale positivity in high-grade undifferentiated pleiomorphic sarcoma (UPS) (*n* = 16). CD47 scale positivity indicators: 0 no staining of tumor cells, 1 + expression in less than 1/3 of tumor tissue, 2 + expression in 1/3 to 2/3 of tumor tissue, and 3 + expression in more than 1/3 of tumor tissue. *p* values were calculated using Mann–Whitney *U* test

First, we have analyzed CD47 abundance in all sarcoma samples (*n* = 55) to evaluate its presence in general. In the treatment-naïve patient cohort, most tumor tissues expressed CD47 (Fig. 2A). Since the tumor grade accounts for one of the most reliable prognostic factors in STS, we evaluated CD47 abundance in G1 + G2 and G3 tumors. We have not observed any significant difference when the entire cohort was analyzed together (Fig. 2B). Next, we evaluated the three most prevalent histological subtypes, LPS, MFS, and UPS, constituting collectively over 80% of the study cohort (Fig. 2C). Surprisingly, the expression levels of CD47 between the G3 and the combined G1 + G2 groups tended to be higher in the G3 group when only LPS, MFS and UPS were collectively examined (Fig. 2D), although this difference did not reach statistical significance (*p* = 0.107) (Fig. 2D). Focusing on LPS, nearly all specimens displayed positivity for CD47, with over 60% of the samples exhibiting moderate-to-diffuse positivity in both G1 + G2 and G3 groups (Fig. 2E–F). Considering LPS as a heterogenous group of tumors, where each subtype has unique characteristics, we have further subdivided LPS. This splitting of LPS revealed that all DDLPS, myxoid LPS (MLPS), and pleiomorphic LPS (PLPS) were CD47 positive (Fig. 2G). Myxofibrosarcomas (MFS) were highly positive for CD47, particularly in the G3 group (Fig. 2H–I). Furthermore, a remarkable proportion of G3 UPS (75%) showed diffuse positivity (Fig. 2J).These findings support CD47 as a promising target for STS therapies.

### CD47 levels demonstrated no correlation with tumor grade, metastases, or mortality

Through our investigation, we hypothesized that CD47 could also provide valuable information about the prognosis and expected outcomes for patients with STS. Given the crucial role of reliable biomarkers in facilitating patient stratification and enabling personalized therapies and targeted interventions based on individual risk profiles, our study sought to systematically investigate the association and prognostic relevance of CD47 on the clinical outcome of patients. First, we investigated the association of CD47 expression with tumor grade, metastases, and death occurrence (Table 2). When analyzing individual levels of CD47 expression (0–3 +) independently, no correlations were identified.

**Table 2:** Chi-square test of independence to assess the association between CD47 expression and grade, metastases, and death

	CD47				*x*^2^	*p* value
	0	1 +	2 +	3 +		
Grade						
G1 + G2	3	2	5	5	4.914	0.178
G3	3	8	6	23		
Metastases presence before sampling						
No	4	8	10	23	1.561	0.668
Yes	2	2	1	5		
Metastases development after sampling						
No	3	6	9	18	0.856	0.836
Yes	1	2	1	5		
Death						
No	5	6	7	18	1.013	0.798
Yes	1	4	4	10		

Considering the importance CD47 expression as an escape mechanism, we also compared extremely high expression of this molecule with other positivity levels (Table 3). However, this division did not show any association with tumor grade, metastasis development and occurrence, or death.

**Table 3:** Chi-square test of independence to assess the association between CD47 expression (CD47 3 + vs CD47 0–2 +) and grade, metastases, and death

	CD47		*x*^2^	*p* value
	0–2 +	3 +		
Grade				
G1 + G2	10	17	2.549	0.110
G3	5	23		
Metastases presence before sampling				
No	22	23	0.004	0.949
Yes	5	5		
Metastases development after sampling				
No	18	18	0.089	0.766
Yes	4	5		
Death				
No	18	18	0.186	0.853
Yes	19	10		

### CD47 may serve as an independent prognostic factor in patients with STS

**Fig. 3:**
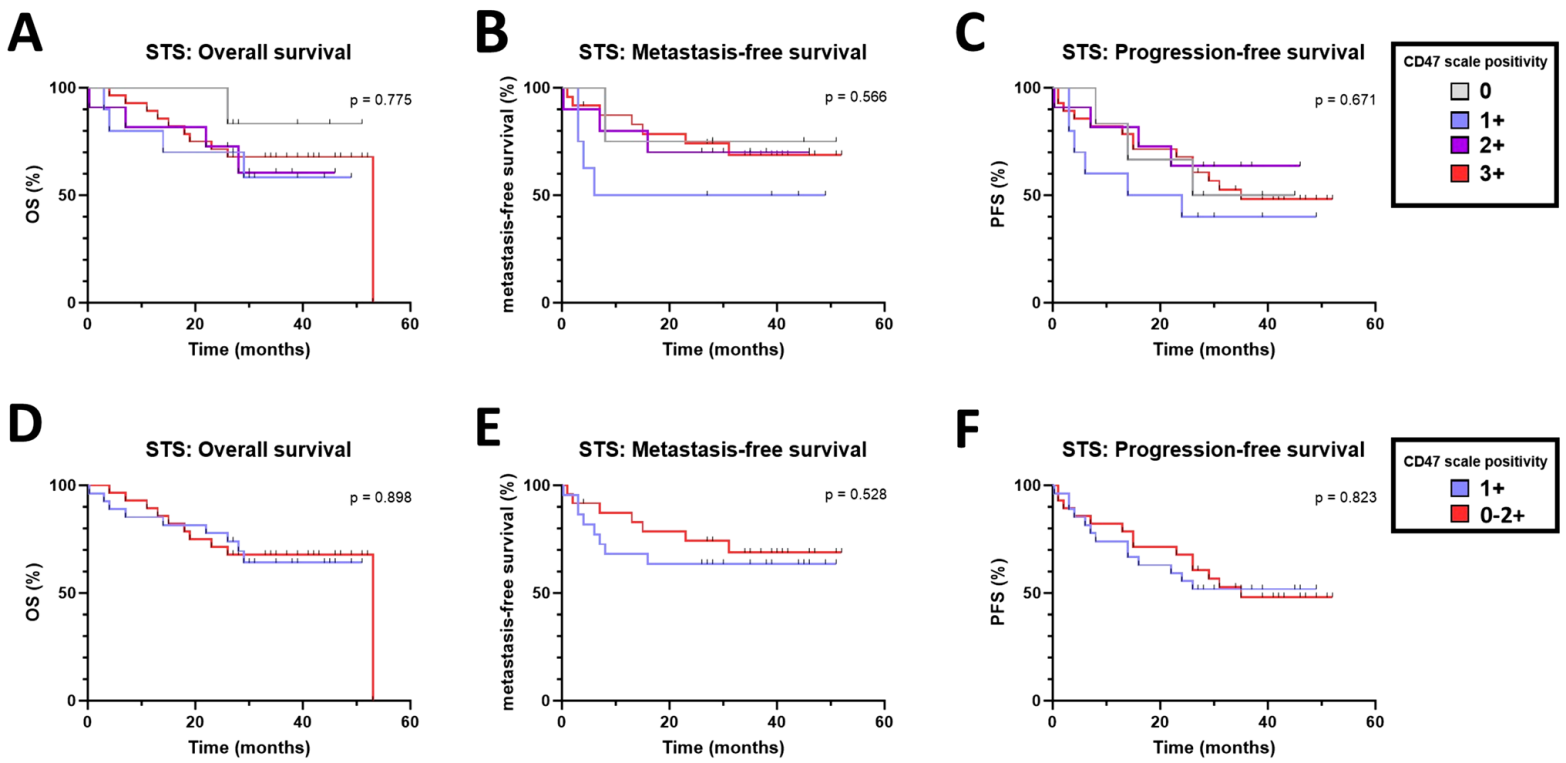
Association between CD47 expression and clinical outcome of patients with soft tissue sarcoma (STS). Kaplan–Meier curves depict the association between CD47 and **A** overall survival (OS), **B** metastasis-free survival, and **C** progression-free survival (PFS) in patients divided according to CD47 scale positivity. Kaplan–Meier curves show **D** OS, **E** metastasis-free survival, and PFS in CD47 0–2 + and CD47 3 + patients. Censored patients have not experienced the event of interest (progression, metastasis, death) by the end of the study period. The small vertical lines serve as a visual cue to underscore the presence of censored observations. *p* values were calculated by log-rank test

Next, we carried out analysis to assess association between CD47 expression and three survival outcomes: OS, metastasis-free survival, and PFS. Our analyses did not reveal any statistically significant differences in terms of OS (Fig. 3A), metastasis-free survival (Fig. 3B), or PFS (Fig. 3C) when patients were divided according to CD47 scale positivity. Additionally, no difference has been observed in OS (Fig. 3D), metastasis-free survival (Fig. 3E), and PFS (Fig. 3F) when we compared CD47 0–2 + and CD47 3 + patients.

Additionally, we used a multivariate Cox proportional hazards model to assess the prognostic relevance of CD47 expression with respect to several clinicopathological variables, including histological subtype (LPS, MFS, UPS and varia), tumor grade, metastases, sex, and age. No prognostic significance of CD47 in the terms of OS (Table 4) has been observed in our study cohort. Interestingly, high abundance of CD47 expression (CD47 3 +) showed a reduced hazard risk of disease progression when compared to CD47 1 + , (*p* = 0.034) (Table 5).

**Table 4:** Cox proportional hazards model assessing the prognostic significance of CD47 expression on overall survival (OS)

OS	HR	*n*	95% CI	*p* value
CD47 expression				
CD47 0 vs CD47 3 +	1.146	6 vs 28	0.076–27.81	0.923
CD47 1 + vs CD47 3 +	1.476	10 vs 28	0.260–7.455	0.642
CD47 2 + vs CD47 3 +	3.052	11 vs 28	0.564–14.43	0.162
Grade				
G2 vs G1	6.001	5 vs 10	0.088–485.3	0.380
G3 vs G1	6.357	40 vs 10	0.746–152.4	0.144
Histology				
UPS vs LPS	1.882	15 vs 22	0.568–6.627	0.303
MFS vs LPS	1.45e-12	10 vs 22	0–inf	> 0.999
Varia vs LPS	8.515	8 vs 22	0.409–123.5	0.116
Metastases				
Yes vs no	4.661	20 vs 35	1.540–16.60	0.010**
Gender				
Female vs male	0.434	26 vs 29	0.078–2.010	0.300
Age	1.101		1.043–1.182	0.002**

Hazard ratios (HR) and 95% confidence intervals (CI) are shown. p values ≤ 0.05 were considered significant

Bold shows statistically significant p values

**Table 5:** Cox proportional hazards model assessing the prognostic significance of CD47 expression on progression-free survival (PFS)

PFS	HR	*n*	95% CI	*p* value
CD47 expression				
CD47 0 vs CD47 3 +	3.437	6 vs 28	0.510–22.73	0.190
CD47 1 + vs CD47 3 +	3.517	10 vs 28	1.065–11.38	0.034*
CD47 2 + vs CD47 3 +	0.833	11 vs 28	0.173–3.035	0.797
Grade				
G2 vs G1	36.54	5 vs 10	3.558–654.9	0.006**
G3 vs G1	2.759	40 vs 10	0.613–20.39	0.236
Histology				
UPS vs LPS	2.931	15 vs 22	1.099–8.304	0.035*
MFS vs LPS	0.151	10 vs 22	0.010–1.091	0.113
Varia vs LPS	0.949	8 vs 22	0.149–4.655	0.952
Gender				
Female vs male	1.243	26 vs 29	0.218–1.359	0.214
Age	2.012		1.002–1.075	0.044*

Hazard ratios (HR) and 95% confidence intervals (CI) are shown. p values ≤ 0.05 were considered significant

Bold shows statistically significant p values

## Discussion

Metastatic sarcoma still presents a major clinical challenge due to the limited effectiveness of existing treatment options in achieving a curative result. Existing therapeutic approaches primarily aim to reduce disease symptoms and extend patient survival, albeit for a relatively short duration, typically spanning only a few months (Meyer & Seetharam 2019). ICI have resulted in achieving lasting complete responses in patients diagnosed with various cancer types, including non-small cell lung cancer, renal cell carcinoma, and metastatic melanoma (Lee et al. 2022). However, there is currently no evidence for routine use of ICI in patients with sarcomas. Despite their efficacy in specific histological subtypes of STS and specific subset of patients, ICI have generally exhibited limited success in the broad sarcoma patient population (Italiano et al. 2022; Keung et al. 2020; Tawbi et al. 2017). Therefore, it is crucial to explore predictive biomarkers and novel potential immune cell targets within the TME of STS.

Most of the research in STS concentrates on T cells. However, increasing attention is given to innate immunity, which offers countless promising targets. This new research direction is even more interesting because macrophages are often the most abundant immune population in STS (Dancsok et al. 2020; Tsagozis et al. 2019). An especially promising immune checkpoint molecule is CD47, which impedes phagocytosis by macrophages and hence protects healthy cells. Many cancer cells exploit this mechanism and overexpress CD47 (Huang et al. 2022).

In our patient cohort consisting of various STS, half of the samples had more than 2/3 of the total tumor area positive on CD47 staining. To offer a more profound insight, we have divided LPS according to their histological subtypes, because we are aware of their considerable differences (Resag et al. 2022). Interestingly, only one WDLPS out of all 22 LPS specimens, did not express CD47. To date, the available data evaluating CD47 expression in STS are rather scarce. The only existing study showed bimodal expression of CD47, where most of the tissue microarrays of sarcoma had either 0% or > 90% positivity in tumor cells. In this study, the range for one category laid between 1 and 89% positivity, which makes the results difficult to compare (Dancsok et al. 2020).

In contrast to the research conducted by Dancsok et al., where approximately 40% of patients exhibited CD47 positivity in a minimum of 1% of tumor cells, our study revealed striking differences. Concerning the MFS histological subtype, a noteworthy 91% of our samples showcased CD47 expression. Furthermore, in the aforementioned study, around 20% of UPS demonstrated a minimum of 1% positivity (Dancsok et al. 2020), while our study showed that 75% of the samples exhibited CD47 expression across a minimum of two-thirds of the tumor area. Notably, our UPS cohort consisted only of high-grade samples, which might have impacted these results. On the other hand, when comparing the CD47 expression between G1 + G2 and G3, no significant differences were observed. However, patients in G1 + G2 groups tended to have lower CD47 expression compared to G3.

In this context, our analysis has not revealed any association between tumor grade and the expression of CD47. To the best of our knowledge, we are the first to compare the differences between CD47 abundance at different tumor grades in STS. Given that we have demonstrated a relatively high expression of CD47 independent of tumor grade, this finding serves as an initial motivation for pursuing further investigations and exploring the potential of targeting the CD47/SIRP-α pathway.

Search for novel prognostic biomarkers in sarcomas is a highly relevant topic. The prognostic relevance of CD47 expression has been described in a number of cancer types. In a study by Huang et al., the authors performed a comprehensive analysis examining the correlation between CD47 expression and OS across 33 cancer types and demonstrated that elevated CD47 expression was indicative of a poorer survival outcome in malignancies, such as pancreatic adenocarcinoma or kidney renal papillary cell carcinoma. Conversely, CD47 expression was associated with a more favorable prognosis in skin melanoma or thyroid carcinoma. (Huang et al. 2022). Another study conducted in non-small cell lung cancer (NSCLC) tumor tissues and cell lines demonstrated that CD47 is overexpressed in these tumors and is significantly associated with clinical staging, lymph node metastases, and distant metastases (Zhao et al. 2016). These findings were supported by an animal study demonstrating that CD47 contributes to the formation and metastasis of T-cell lymphoma in vivo (Kitai et al. 2021). It is noteworthy to acknowledge that CD47 blockade has demonstrated remarkable success in the treatment of hematological malignancies (Hai et al. 2022).

In a single retrospective study by Tanaka et al., the authors investigated the prognostic relevance of SIRPα and CD47 expression within the TME among patients diagnosed with myeloid sarcoma. Although no significant prognostic distinctions were observed between patients with SIRPα-positive neoplastic cells (nSIRPα) and those exhibiting SIRPα expression on non-neoplastic stromal cells in the TME (miSIRPα), individuals with CD47-positive myeloid sarcoma experienced notably prolonged OS compared to their counterparts with CD47-negative myeloid sarcoma (Tanaka et al. 2024).

Several other studies in solid tumors have yielded inconclusive results, failing to demonstrate any discernible clinical significance. Specifically, no clinical significance was observed in muscle-invasive bladder cancer or endometrial carcinoma (Myint et al. 2023; Sercan et al. 2022). We have not observed any association of CD47 expression with metastases occurrence or death. In addition, CD47 positivity did not influence OS, PFS, and metastasis-free survival. However, high CD47 expression showed reduced risk of disease progression when compared to low CD47 expression in the multivariate Cox proportional hazards model. Similarly, in Dancsok et al. study lower CD47 abundance was associated to enhanced risk of disease progression in translation-associated sarcoma when Cox proportional hazards model was employed. In their analysis, the univariable Cox proportional hazards model for OS revealed the prognostic significance of CD47 in some histological subtypes, including UPS (Dancsok et al. 2020).

Given the high expression of CD47 in STS, focusing on this pathway could be a promising therapeutic approach. Anti-CD47/SIRP-α inhibitors have been investigated in preclinical studies and clinical trials across hematologic and solid cancers (Li et al. 2021). Taking into consideration the limited therapeutic efficacy of current treatment strategies, anti-CD47 treatment might hold a potential for patients with STS. We have previously shown in vitro comparable production of pro-inflammatory cytokines after either anti-PD1 or anti-CD47 blockade in 10 STS patients (Ozaniak et al. 2022), a novel potential approach for STS treatment. Notably, in vitro blockade of CD47 on human LMS cell lines and in LMS mice model resulted in increased phagocytosis and reduced tumor size respectively (Edris et al. 2012). In light of this, a clinical trial is currently ongoing, investigating the synergistic effects of doxorubicin in combination with TTI-621, an anti-CD47 therapeutic agent, in patients with unresectable or metastatic high-grade LMS (Chawla et al. 2022).

High CD47 expression was associated with tumor mutation burden and microsatellite instability in certain cancers, which are both associated with response to ICI (Huang et al. 2022). This should be further investigated in STS because it could further encourage combinatorial and patient-tailored immunotherapeutic approaches. Moreover, CD47 expression influenced response to adjuvant chemotherapy in patients with ovarian cancer (Brightwell et al. 2016), and thus CD47 might serve as a predictive marker as well.

This study has several limitations. Owing to the rarity of the studied tumors, our cohort exhibits a relatively heterogeneous distribution of histological subtypes, with certain histologies represented only once. Furthermore, it is crucial to recognize the disparity in the distribution of low-grade and high-grade tumors. Lastly, given that our study exclusively includes primary tumors, we were unable to assess potential variations in CD47 expression within metastatic lesions. In conclusion, CD47 molecule might be a powerful prognostic and predictive factor in various cancers, although its potential in STS remains to be clarified. Currently, CD47 molecule is a worthy candidate for future immunotherapeutic drugs. Our results suggest that while CD47 expression is prevalent in various sarcoma subtypes, its impact on survival outcomes and prognostic relevance may be limited. Further investigations are needed to fully explore CD47 role in STS and its potential targeting.

## Data Availability

The authors are willing to provide their data upon reasonable request to the corresponding author for the purpose of facilitating scientific collaboration and further exploration of the study findings.
